# TNFAIP8L3 regulation of the TGF-β signaling pathway affects the proportion of macrophages during tumor antigen presentation and affects the prognosis of ovarian cancer

**DOI:** 10.3389/fmolb.2025.1566363

**Published:** 2025-04-24

**Authors:** Chaomin Yue, Xiao Zheng, Yuqing Ye, Danyang Li, Yanhong Zhou

**Affiliations:** ^1^ Department of Obstetrics and Gynaecology, Guangzhou Women and Children’s Medical Center, Guangzhou Medical University, Guangzhou, China; ^2^ Department of General Surgery, Yueyang Hospital of Integrated Traditional Chinese and Western Medicine, Shanghai University of Traditional Chinese Medicine, Shanghai, China; ^3^ School of Medicine, Dentistry and Nursing, University of Glasgow, Glasgow, United Kingdom; ^4^ Department of Pharmacology, College of Pharmacy, Harbin Medical University, Harbin, China

**Keywords:** TNFAIP8L3 (TIPE3), TGF-β signaling pathway, ovarian cancer, antigen presentation, TCGA database

## Abstract

**Background:**

Ovarian cancer is a serious disease that is a danger to a woman’s life and health and is currently being extensively studied worldwide. TNFAIP8L3, a member of the tumor necrosis family, plays a significant role in tumor immune regulation; however, its role in ovarian cancer has not been fully studied and reported.

**Method:**

This study used data from 381 cases of ovarian cancer (OVCA) from The Cancer Genome Analysis (TCGA)-OV to analyze the clinical phenotype and immune phenotype of TNFAIP8L3. Pearson correlation coefficients and protein–protein interaction (PPI) network analyses were used to identify potential biological functions and the genes co-expressed with TNFAIP8L3. The Gene Set Enrichment Analysis (GSEA) method was used to evaluate the relevant regulatory pathways of TNFAIP8L3. Gene Set Variation Analysis was performed to evaluate the proportions of 24 immune cell types in OVCA. Finally, a nomogram and multivariate COX regression analysis were performed to prove the independent prognostic value of TNFAIP8L3 in OVCA.

**Results:**

A total of 181 genes were co-expressed with TNFAIP8L3, including 163 positively correlated and 18 negatively correlated genes. The co-expressed genes associated with TNFAIP8L3 were significantly enriched in biological processes such as cell activation involved in immune response and leukocyte activation involved in immune response. Pathway analysis of TNFAIP8L3 further revealed the association of TNFAIP8L3 with the TGF-β signaling pathway and antigen processing and presentation pathways, especially with macrophages and neutrophils in the antigen presentation process. Moreover, the expression of TNFAIP8L3 was significantly high in OVCA and other solid tumors to function as an independent risk factor for the prognosis of OVCA, with important research value for the prognosis of patients with OVCA.

**Conclusion:**

Those findings indicate that TNFAIP8L3 is correlated with antigen processing and presentation pathways in macrophages and neutrophils and affects prognosis and immune infiltration in OVCA.

## Introduction

Ovarian cancer (OVCA) ranks third in terms of annual incidence among tumors affecting the female reproductive system, with an increasing yearly trend. It exerts the highest mortality rate among malignant tumors of the female genital tract, posing a serious threat to women’s health. In 2022, China reported 57,200 new cases of OVCA and 27,200 associated deaths, surpassing the global average incidence and mortality rates of, respectively, 5.59 and 2.45 per 100,000 (Saso et al., 2024). Over recent years, innovative treatment modalities, including targeted therapy, immunotherapy, and antibody-drug conjugate therapy, have offered hope for patients suffering from gynecological cancers. Current diagnostic techniques involve serum alpha-fetoprotein testing, ultrasound examinations, and CT scans; however, these methods are associated with a high rate of misdiagnosis (Massariol Pimenta et al., 2024; Clair et al., 2025). To enhance the precision of early detection, identification of the causes of OVCA and effective prognostic biomarkers are crucial (Tonti et al., 2024; Lazaridis et al., 2024).

TNFAIP8L3/TIPE3 is a member of the tumor necrosis factor-alpha (TNF)-α-induced protein 8 (TNFAIP8/TIPE) family. It has a key role in regulating inflammatory responses, maintaining immune homeostasis, and affecting cancer development (Gu et al., 2020). Earlier published studies have shown that the TNFAIP8L3 protein can promote cell viability and induce drug resistance, consequently promoting the development and progression of cancer (Fayngerts et al., 2014; Day et al., 2017). Elevated TNFAIP8L3 expression has been observed in various cancers, including colorectal, non-small cell lung, breast, esophageal, stomach, and malignant glioma (Cui et al., 2015). TNFAIP8L3 can trigger the phosphatidylinositol 3-kinase/protein kinase B (PI3K) signaling pathway, thereby enhancing the proliferation and migration of gastric cancer cells (Gao et al., 2020). In breast cancer, TIPE3 also facilitates metastasis through the activation of AKT and nuclear factor-kappa B (NF-κB) signaling pathways (Lian et al., 2017). TNFAIP8L3 also facilitates the progression of malignant glioma by suppressing phosphorylation of p38 (Yuan et al., 2019). These factors make it important to investigate the function of TNFAIP8L3 in OVCA detailed in these reports and assess its potential as a predictive biomarker.

## Method

### Ovarian cancer data acquisition

The gene expression matrix and clinical follow-up data of tumor tissues of patients with OVCA were obtained from The Cancer Genome Analysis (TCGA) data (https://cancergenome.nih.gov/), including 381 OVCA samples and no adjacent tissue samples. Relevant corresponding clinical data were also obtained from the XENA database. The clinical characteristics of the patients included gender, survival status, survival time, Tumor, Lymph node, metastasis stage (TNM), etc. All raw data used in this study are available in the database.

### TNFAIP8L3 gene co-expression analysis

A co-expression analysis of protein editing genes was performed in the entire TCGA-OV matrix based on the transcript per million (TPM) expression of TNFAIP8L3. For the analysis, Pearson correlation coefficient analysis was performed. Correlation values >0.4 showed a significant positive correlation with the TNFAIP8L3 gene. The coefficient Cor <−0.26 (Basha et al., 2024) was significantly negatively correlated with the TNFAIP8L3 expression level; the expression patterns of these genes are considered to be similar to the TNFAIP8L3 gene. Genes exhibiting similar expression patterns are involved in the same biological functions. Thus, the potential biological functions of TNFAIP8L3 can be discovered by analyzing the co-expressed genes of TNFAIP8L3.

### TNFAIP8L3 co-expression gene Gene Ontology (GO) enrichment analysis

Functional enrichment analysis of TNFAIP8L3 co-expressed genes was performed through GO using the “clusterProfiler” package (v4.4.4) in the R environment (v4.2.1) to analyze the biological processes, cellular composition, and molecular functions of genes co-expressed with TNFAIP8L3. Adjusted *p* < 0.05 was considered a significant enrichment. The list of relevant co-expressed genes is presented in Supplementary Table 1.

### Gene set enrichment analysis

Samples in the TCGA-OV TPM cohort were categorized into two groups based on the expression of TNFAIP8L3. We predicted the function and role of TNFAIP8L3 by using the GSEA 4.1.0 software and performed gene enrichment analysis (GSEA) on patients with OVCA in different TNFAIP8L3 expression groups using the *c2.cp.kegg.v7.0.symbols.gmt* dataset as the functional gene set in the Molecular Signature Database (MsigDB). The number of iterations was set to 1,000, and other parameters were set to default values.

### TNFAIP8L3 protein–protein interaction (PPI) network

The PPI network of the co-expressed genes of TNFAIP8L3 in Supplementary Table 2 was obtained and analyzed through the STRING database (Franceschini et al., 2013) (http://string-db.org/), and a comprehensive score >0.4 was considered significant. Next, the open-source software Cytoscape 3.7.1 (Smoot et al., 2011) was used to visualize the biological network of protein interactions. Then, the PPI network and significance module were established using the following criteria: MCODE score >5, cutoff value = 2, node score cutoff value = 0.2, max depth = 100, and k-score = 2.

### The z-score evaluation of the TNFAIP8L3-related biological process

The z-score, an algorithm proposed by Lee et al. (2008), reflects the activity of given pathways by integrating feature gene expressions. Gene sets containing the genes referring to *ECM receptor interaction, focal adhesion, JAK-STAT signaling pathway, TGF-β signaling pathway, cytokine-cytokine receptor interaction, antigen processing and presentation, chemokine signaling pathway,* and *T cell receptor signaling pathway* were subjected to the z-score algorithm implemented in the R package GSVA. The pathway genes are listed in Supplementary Table 3.

### TNFAIP8L3 immune microenvironment analysis

GSVA analysis of 24 immune cell types was performed to evaluate the proportion of immune cells in the immune microenvironment in OVCA.

### TNFAIP8L3 prognostic models

A nomogram was generated based on the results of multivariate TNFAIP8L3 COX for personalized prediction of patient prognosis analysis, and 1-year, 2-year, 3-year, and 5-year overall survival (OS) rates were predicted using the rms package. Information loss was minimized through a reverse reduction selection process to incorporate independent prognostic factors into the final nomogram model meeting the Akaike information criterion. The predictive performance and discriminative ability of the nomogram were respectively evaluated through Harrell’s conformance index (C-index) and calibration curves.

## Results

### TNFAIP8L3 co-expression analysis

**FIGURE 1:**
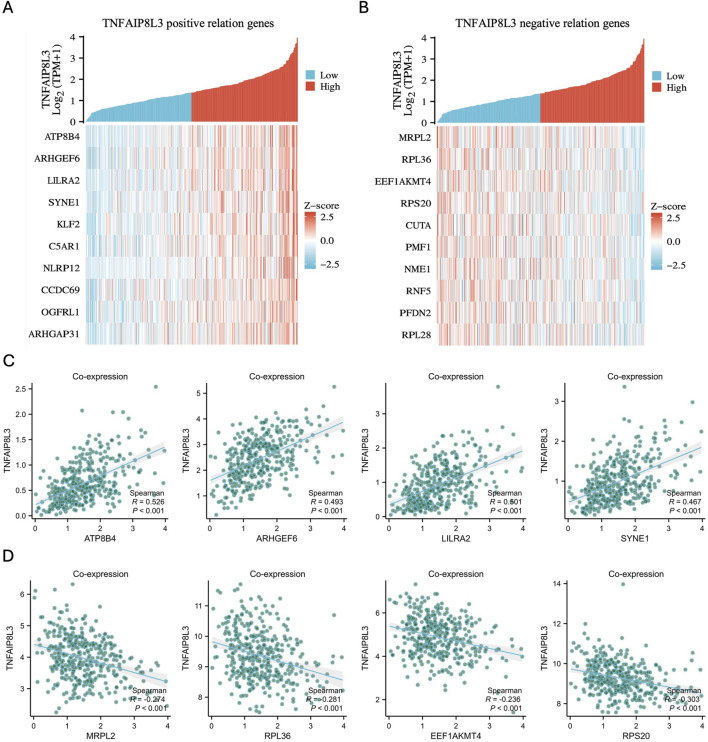
The result of co-expression analysis. **(A)** The heat map of the 10 positive genes correlated with TNFAIP8L3. *p < 0.05.* **(B)** The heat map of the 10 negative genes correlated with TNFAIP8L3. **(C)** The correlations between ATP8B4, ARHGEF6, LILRA2, SYNE1 expression, and TNFAIP8L3 were positive. **(D)** The correlations between MRPL2, RPL36, EEF1AKMT4, and RPS20 expression and TNFAIP8L3 were negative.

The co-expression analysis of TNFAIP8L3 yielded 181 TNFAIP8L3-associated co-expressed genes, with 163 positively correlated and 18 negatively correlated. The top 10 genes with positive correlation coefficients and the top 10 genes with negative correlation coefficients are presented in heat maps (Figure 1). The genes ATP8B4, ARHGEF6, LILRA2, SYNE1, KLF2, C5AR1, NLRP12, CCDC69, OGFRL1, and ARHGAP31 were positively correlated (Figure 1A), while MRPL2, RPL36, EEF1AKMT4, RPS20, CUTA, PMF1, NME1, RNF5, PFDN2, and RPL28 were negatively correlated with TNFAIP8L3 expression (Figure 1B). The relevant gene expression levels are presented as a scatter plot, as shown in Figures 1C, D.

### TNFAIP8L3 biological function

**FIGURE 2:**
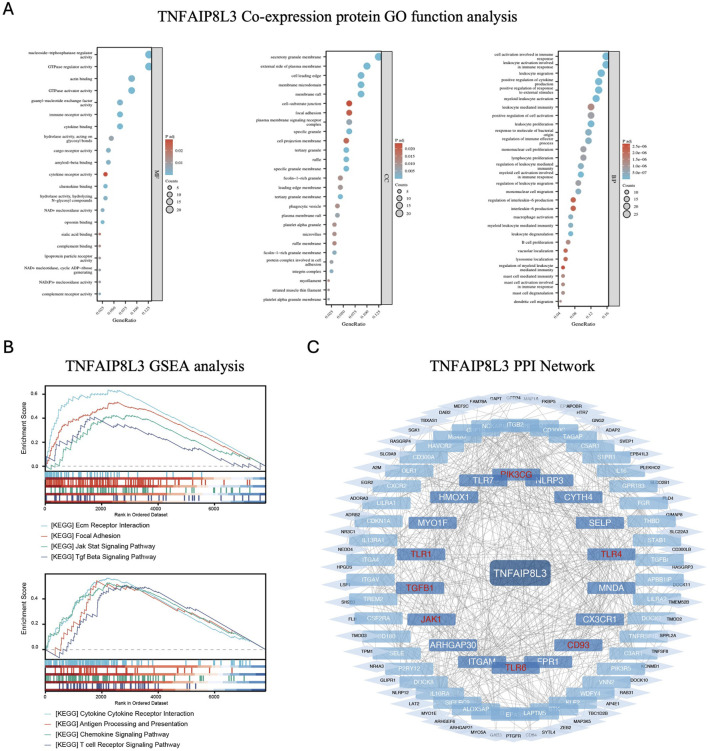
Enrichment analysis of TNFAIP8L3 in ovarian cancer (OVCA). **(A)** Molecular function, cellular component, and biological process enrichment analyses associated with TNFAIP8L3-related genes. **(B)** The results of enrichment analysis from Gene Set Enrichment Analysis. **(C)** A network presenting TNFAIP8L3 and its potential co-interaction proteins.

Functional enrichment on the 181 co-expressed genes mentioned above revealed the enrichment of these genes in biological processes such as cell activation and leukocyte activation involved in immune response, cellular components, including secretory granule membrane and external side of the plasma membrane, and molecular functions, including nucleoside triphosphatase regulator activity and GTPase regulator activity (Figure 2A). In addition, in terms of pathways, these genes were significantly enriched in *ECM receptor interaction, focal adhesion, JAK-STAT signaling pathway, TGF-β signaling pathway, cytokine-cytokine receptor interaction, antigen processing and presentation, chemokine signaling pathway,* and *T cell receptor signaling pathway*. These findings suggest the role of TNFAIP8L3 in the proliferation and immunological regulation of OVCA cells (Figure 2B).

Next, the PPI network of these co-expressed proteins was successfully constructed through *Cytoscape* (Figure 2C). The proteins in the PPI network close to and closely linked to TNFAIP8L3 are the potential interacting proteins; among these, PIK3CG, TLR4, CD93, TLR6, JAK1, TGF-β1, TLR1, and other proteins are considered to interact closely with TNFAIP8L3, suggesting the potential functions of these proteins and ultimately leading to poor prognosis of patients with OVCA. It is believed that the interaction between TNFAIP8L3 and these proteins could play a crucial role in the progression of ovarian cancer. For instance, the binding of TNFAIP8L3 with PIK3CG may influence the PI3K/AKT signaling pathway, which is known to be involved in cell growth and survival. Similarly, the interaction with TLR4 and TLR6 could indicate a role in the innate immune response, which is often dysregulated in cancer. The association with JAK1 might implicate a role in cytokine signaling, while the connection with TGF-β1 could suggest involvement in cell proliferation and differentiation. Understanding these interactions could provide valuable insights into the molecular mechanisms underlying the disease and potentially identify new therapeutic targets for improving patient outcomes.

### Pan-cancer pathway analysis of TNFAIP8L3 in solid tumors

**FIGURE 3:**
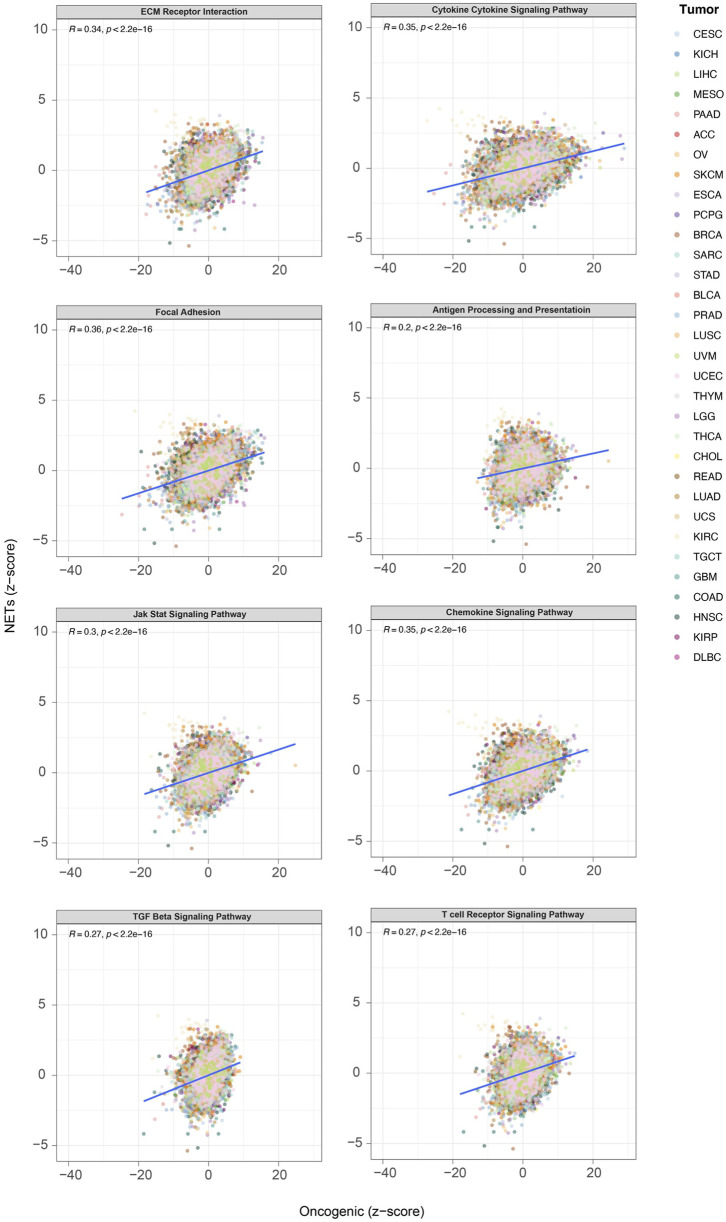
The outcomes of the pan-cancer pathway analysis of TNFAIP8L3.

According to the above results, TNFAIP8L3 is related to cell proliferation and immune-related pathways and interacts with key proteins in these pathways. However, these data were obtained only from 381 samples of OVCA in TCGA. We also conducted a heterogeneity analysis of TNFAIP8L3 and key pathways in other solid tumors to enhance the reliability of these conclusions. The level of consistency analysis was evaluated and scored using the Z-score of GSVA. The results showed the involvement of TNFAIP8L3 in all solid tumors in *ECM receptor interaction, focal adhesion, JAK-STAT signaling pathway, TGF-β signaling pathway, cytokine-cytokine receptor interaction, antigen processing and presentation, chemokine signaling pathway,* and *T cell receptor signaling pathway.* The significant regulatory relationships are presented in Figure 3.

### Immune microenvironment analysis of TNFAIP8L3

**FIGURE 4:**
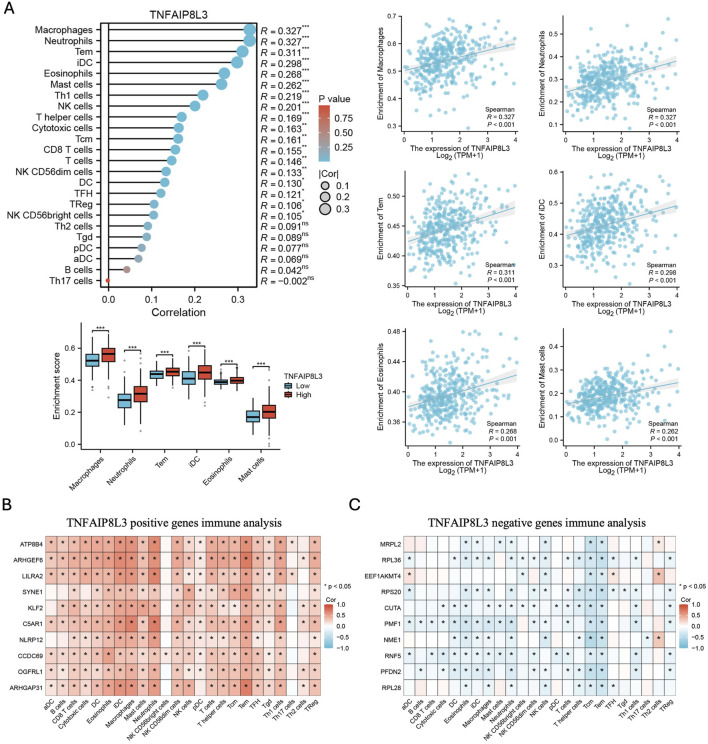
The analysis between TNFAIP8L3 expression and immune infiltration. **(A)** Correlation between the level of TNFAIP8L3 expression and the relative abundances of 24 immune cell types. **(B)** The heat map of the genes positively correlated with TNFAIP8L3 and 24 immune infiltration cell types. **(C)** The heat map of the negatively correlated genes of TNFAIP8L3 and 24 immune infiltration cell types.

A comprehensive cohort analysis revealed that TNFAIP8L3 may potentially affect the heterogeneity of the immune microenvironment; therefore, we used the GSVA algorithm and evaluated the relative proportions of 24 immune cell types in the immune microenvironment in detail and performed correlation analysis based on the level of TNFAIP8L3 expression. Indeed, TNFAIP8L3 was observed to be associated with macrophages and neutrophils (Figure 4A). Based on the above-mentioned pathway analysis, we hypothesize that TNFAIP8L3 is involved in the processing and presentation of tumor antigens, enhancing antitumor immunity. Concurrently, the genes co-expressed with TNFAIP8L3 were applied for the correlation analysis of immune cells. The genes positively correlated with TNFAIP8L3 were also found to be significantly positively correlated with neutrophils and macrophages and consistently obtained negatively correlated groups (Figures 4B, C).

### Clinical phenotype and pan-cancer heterogeneity analysis of TNFAIP8L3

**FIGURE 5:**
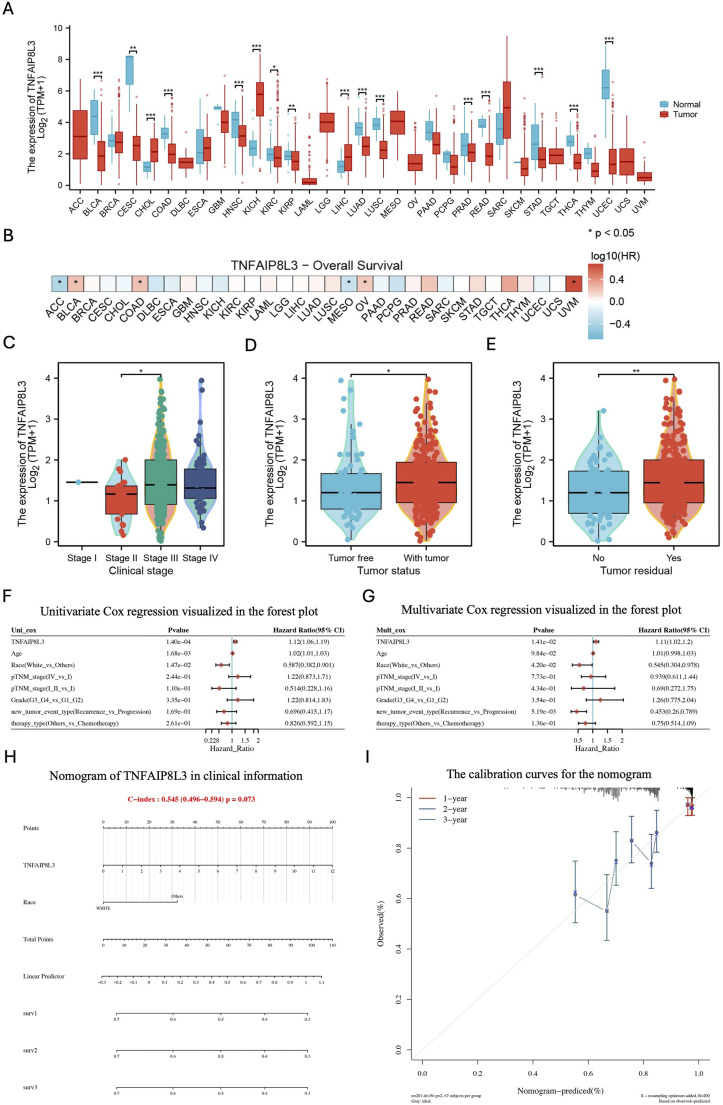
Clinical phenotype and pan-cancer heterogeneity analysis of TNFAIP8L3. **(A)** The differential expression of TNFAIP8L3 in cancer and paracancer tissues in pan-cancer analysis. **(B)** Association of TNFAIP8L3 with overall survival in pan-cancer analysis. **(C–E)** Association of the TNFAIP8L3 and different clinicopathologic characteristics. **(F)** Unitivariate Cox regression in the forest plot. **(G)** Multivariate Cox regression in the forest plot. **(H)** The nomogram of TNFAIP8L3 based on clinical information. **(I)** The calibration curves of TNFAIP8L3. Note: ACC, adenoid cystic carcinoma; BLCA, bladder cancer; BRCA, breast cancer; CESC, cervical squamous cell carcinoma; CHOL, cholangiocarcinoma; DLBC, diffuse large B-cell lymphoma; ESCA, esophageal carcinoma; GBM, glioblastoma; HNSC, head and neck squamous cell carcinoma; KICH, kidney chromophobe; KIRC, kidney renal clear cell carcinoma; KIRP, kidney renal papillary cell carcinoma; LAML, acute myeloid leukemia; LGG, low-grade glioma; LIHC, liver hepatocellular carcinoma; LUAD, lung adenocarcinoma; LUSC, lung squamous cell carcinoma; MESO, mesothelioma; OV, ovarian cancer; PAAD, pancreatic adenocarcinoma; PCPG, pheochromocytoma and paraganglioma; PRAD, prostate adenocarcinoma; READ, rectum adenocarcinoma; SARC, sarcoma; SKCM, skin cutaneous melanoma; STAD, stomach adenocarcinoma; TGCT, testicular germ cell tumors; THCA, thyroid carcinoma; THYM, thymoma; UCES, uterine corpus endometrial carcinoma; UCS, uterine carcinosarcoma; UVM, uveal melanoma.

Pathway analysis revealed the relationship of TNFAIP8L3 to the pathway of OVCA progression. As the corresponding OVCA adjacent tumor expression data were lacking in TCGA-OV, we performed TNFAIP8L3 tumor difference analysis in all solid tumors. Among these, TNFAIP8L3 was significantly upregulated in kidney chromophobe (KICH), kidney renal clear cell carcinoma (KIRC), cholangiocarcinoma (CHOL), and liver hepatocellular carcinoma (LIHC) tumor tissues (Figure 5A). In addition, the prognostic hazard risk (HR) ratio of TNFAIP8L3 in urothelial bladder carcinoma (BLCA), colon adenocarcinoma (COAD), OVCA, and uveal melanoma (UVM) is >1, making it a prognostic risk factor for the above-mentioned tumors (Figure 5B).

In OVCA, TNFAIP8L3 was associated with the clinical stage, tumor status, and tumor residual. TNFAIP8L3 was significantly overexpressed in advanced clinical stages and was significantly associated with residual tumor status and positive resection margin rate (Figures 5C–E). The above findings thus prove that TNFAIP8L3 is associated with poor prognosis of OVCA; however, whether TNFAIP8L3 can be independent of clinical indicators and exert its independent prognostic value still needs investigation. Therefore, follow-up data combined with clinical indicators, including clinical stage, gender, and age, were employed to prove the independent prognostic value of TNFAIP8L3 in OVCA. The risk ratio in the multivariate COX analysis of TNFAIP8L3 was 1.11, which indicates an independent prognostic value (Figures 5F, G). Finally, TNFAIP8L3 was incorporated into the clinical prognosis prediction nomogram model and proved that it can predict the clinical prognosis of OVCA patients for 1 year, 3 years, and 5 years (Figures 5H, I).

## Discussion

Our findings indicate that TNFAIP8L3 is associated with both the JAK/STAT and transforming growth factor-beta (TGF-β) signaling pathways and plays a role in the development and progression of OVCA by modulating these pathways. The Janus kinase/signal transducer and activator of transcription (JAK/STAT3) signaling pathway is implicated in numerous critical biological processes, including cell growth, differentiation, apoptosis, and immune regulation (O'Shea et al., 2013). TNFAIP8L3 has been reported to enhance lung cancer cell proliferation, survival, and migration by activating the STAT-3 signaling pathway (Bordoloi et al., 2022). Our analysis led to similar conclusions. In the context of OVCA, TIPE3 is implicated in the regulation of the JAK/STAT signaling pathway, suggesting that the mechanism through which TIPE3 facilitates the progression of OVCA may be potentially linked to the STAT-3 signaling pathway-mediated enhanced cell proliferation, survival, and migration. Nevertheless, it is imperative to devise experiments for further detailed investigation to elucidate the precise mechanism of action of TNFAIP8L3.

The TGF-β signaling pathway is a canonical pathway that regulates cell proliferation and differentiation, and disruptions in TGF-β function can facilitate tumor growth (Luo et al., 2025). It has been observed that the degradation of SMAD7 is predominantly facilitated through the TGF-β/SMAD signaling pathway regulation, thereby propelling the advancement of OVCA (Zhong et al., 2024). TIPE2, a member of the same family, suppresses OVCA metastasis and epithelial–mesenchymal transition by interacting with SMAD2, indicating that our findings need to be experimentally validated for the role of TIPE3 in promoting cancer (Tang et al., 2024).

Whether TNFAIP8L3 directly regulates these pathways or is simply related to their activity remains to be fully elucidated. Our research suggests that TNFAIP8L3 is associated with both the JAK/STAT and TGF-β signaling pathways and influences the development and progression of ovarian cancer (OVCA) by modulating these pathways. The JAK/STAT3 signaling pathway is crucial for various biological processes such as cell growth, differentiation, apoptosis, and immune regulation. Previous studies have reported that TNFAIP8L3 enhances lung cancer cell proliferation, survival, and migration by activating the STAT-3 signaling pathway. Our analysis aligns with these findings, indicating that in the context of OVCA, TNFAIP8L3 may regulate the JAK/STAT signaling pathway, potentially through the STAT-3 signaling pathway-mediated enhancement of cell proliferation, survival, and migration. However, further experiments are necessary to confirm the precise mechanism of action of TNFAIP8L3.

The TGF-β signaling pathway is a well-established pathway that regulates cell proliferation and differentiation, and disruptions in its function can contribute to tumor growth. It has been observed that the degradation of SMAD7 is facilitated primarily through the TGF-β/SMAD signaling pathway regulation, which promotes the progression of OVCA. Additionally, TIPE2, a related family member, suppresses OVCA metastasis and epithelial–mesenchymal transition by interacting with SMAD2. These findings highlight the need for experimental validation to confirm the role of TNFAIP8L3 in promoting cancer.

The TNFAIP8 family participates in inflammation, immunity, and cancer. Members of this family are expressed in various human cancer cell lines, with TIPE1 being particularly significant in secretion and carcinogenesis (Lou and Liu, 2011). Furthermore, the mechanism of action of TIPE3 remains to be thoroughly investigated. Our findings indicate a future research direction, suggesting the possible association of TIPE3 with inflammatory and immune-related signaling pathways, including chemokine signaling and T-cell receptor signaling.

Immune checkpoint therapy is a transformative approach in cancer treatment that augments the capacity of the immune system to inhibit or eliminate tumor cells through the modulation of immune pathways. The landmark discovery of the cytotoxic T lymphocyte-associated protein 4 (CTLA-4/B7-1) and programmed cell death ligand-1/programmed cell death protein-1 (PD-L1/PD-1) interactions are pivotal advancements in tumor immunotherapy, propelling extensive research endeavors into immune checkpoints. Over the past decade, immune checkpoint inhibitors have emerged as a cornerstone of cancer treatment, forming the fourth pillar alongside chemotherapy/targeted therapy, radiation therapy, and surgery. In addition to CTLA-4 and PD-1, the exploration of numerous novel immune regulatory molecules is ongoing for lymphocyte-activation gene 3 (LAG-3), T cell immunoglobulin and mucin-domain containing-3 (TIM-3), T cell immunoglobulin and immunoreceptor-tyrosine-based inhibitory motif domain (TIGIT), inducible T cell costimulator (ICOS), 4-1BB, and OX-40 (Sharma et al., 2023; Wykes and Lewin, 2018). In addition, the application of CAR-T cell therapy in solid tumor tumors is gradually being studied, and the use of CAR-T in the treatment of B-cell lymphoma has been deeply reported, which provides a new direction for the treatment of ovarian cancer (Li et al., 2025).

The interaction between PD-1 and PD-L1 negatively regulates T-cell proliferation, tumor cytotoxicity, and cytokine secretion, simultaneously augmenting the regulatory T-cell (Treg) population. These dynamics serve to preserve self-tolerance and facilitate cancer progression (Zou and Chen, 2008; Hamid et al., 2013; Freeman et al., 2000; Francisco et al., 2009). The anti-PD-1 antibody nivolumab offers clinical benefits to patients with advanced OVCA, particularly those who have not responded to chemotherapy or radiotherapy (Taneja, 2012). During tumor elimination, the interaction of CD80 and CD86 on antigen-presenting cells with CD28 on T cells regulates T-cell activation, thereby initiating T-cell proliferation (Tse et al., 2014). The binding of CTLA-4, a homolog of CD28, with CD80/CD86 generates inhibitory signals that suppress the immune response (Chambers et al., 2001). The binding ratio of CD28 or CTLA-4 to CD80/CD86 dictates the activation or suppression of T cells (Krummel and Allison, 1995). Ipilimumab, a CTLA-4 inhibitor, exhibits potent antitumor efficacy (Hodi et al., 2003).

Our findings indicate that in addition to PD-1 and CTLA4 immune checkpoints, other immune checkpoints, including CD274 and LAG3, play a significant role in the prognosis of OVCA. These findings imply that exploring the therapeutic potential of these immune checkpoints could lead to identifying innovative treatment strategies within the realm of immunotherapy. Further research into these checkpoints in terms of expression patterns and functional roles in OVCA could provide insights into the mechanisms driving tumor progression and immune evasion. Understanding these processes is the key to developing combination therapies targeting multiple checkpoints simultaneously, potentially improving the efficacy of immunotherapy. Identifying biomarkers associated with these checkpoints could also aid in patient categorization, allowing more personalized and effective treatment plans.

The study acknowledges several limitations. The samples analyzed were sourced from the TCGA public database and may not comprehensively represent the status of TNFAIP8L3 in OVCA. Subsequent studies should aim to increase the sample size to validate the expression of TNFAIP8L3 in OVCA and its correlation with clinicopathological characteristics. Moreover, in this study, only the biological function and clinical application value of TNFAIP8L3 were examined using clinical samples. Therefore, it is necessary to incorporate cell-based experiments into future investigations to fully understand the impact of TNFAIP8L3 on the proliferation of OVCA cells.

This study holds significant importance. First, this is the first to report the elevated expression of TNFAIP8L3 in OVCA and its correlation with the proliferation capabilities of OVCA cells, offering new insights into the etiology of the disease. Second, the study introduces a novel concept for the precision medicine approach to treating OVCA. Suppressing TNFAIP8L3 expression could pave the way for personalized therapy, potentially enhancing the rates of survival and quality of life for patients with this condition. Future research should attempt to identify the precise role of TNFAIP8L3 in the progression of OVCA and its interaction with other signaling pathways. Accordingly, targeted therapies against TNFAIP8L3 could be pursued for development, with clinical trials to assess their effectiveness and safety through clinical trials.

TNFAIP8L3, as a gene product, exhibits distinct yet overlapping roles in oncolytic viruses (OVCA) compared to its homologs in other cancer contexts. In the context of OVCA therapy, specifically in studies on glioblastoma multiforme (GBM), TNFAIP8L3 has been implicated in the polarization of myeloid cells toward an M2 phenotype (Wang et al., 2024), which is predominantly located in the peritumoral area. These TNFAIP8L3-high myeloid cells participate in TNF-α signaling and inflammatory responses via NF-κB, suggesting a potential antitumor role. The expression of TNFAIP8L3 in this setting may contribute to recruiting these cells into the GBM microenvironment, facilitating antitumor actions.

In contrast, in other cancer types, homologs of TNFAIP8L3 or related proteins may have different functions. For instance, TNFAIP8, a closely related protein, has been found to be highly expressed in various malignancies such as breast (Lian et al., 2017), prostate (Padmavathi et al., 2018), and lung (Li et al., 2021) cancers. TNFAIP8 functions as a negative regulator of cell apoptosis (Yuan et al., 2019), playing a crucial role in tumor progression by inhibiting caspase activity and leading to the inactivation of caspase-3 and caspase-8. High expression of TNFAIP8 has been shown to promote tumor growth and migration.

While TNFAIP8L3 in the context of OVCA therapy appears to be associated with antitumor immunity, TNFAIP8 in other cancers exhibits oncogenic properties by inhibiting apoptosis and promoting tumor progression. This highlights a functional divergence between TNFAIP8L3 in the OVCA setting and its homologs in other cancer types, where TNFAIP8 may act more directly in tumor cell survival and proliferation.

However, it is important to note that the exact homologs and their interactions in different cancer contexts can vary, and further research is needed to fully elucidate the specific roles of TNFAIP8L3 and related proteins in various types of cancer and their responses to therapeutic interventions. With in-depth studies into the treatment of solid tumors, scientists have discovered numerous new treatment methods. The research breakthroughs of dendritic cell vaccines for treating solid tumors (Zhu et al., 2025) and multifunctional drug delivery systems for magnetic resonance (MR) imaging (Lai et al., 2025) are of landmark significance.

In summary, the study revealed that TNFAIP8L3 is overexpressed in ovarian tumors and is linked to the growth potential of these cells. This finding presents a new target and a novel approach to the treatment of ovarian cancer. Subsequent research should focus on expanding the number of samples, exploring the mechanisms in greater depth, and creating specific treatments for TNFAIP8L3, with the goal of improving outcomes for patients with ovarian cancer.

## Conclusion

TNFAIP8L3 is highly expressed in patients with OVCA and serves as an independent prognostic risk factor. Its effect on OVCA can be mainly attributed to its regulatory effects on TGF-β and tumor antigen presentation processes within the tumor microenvironment. The mechanisms need to be studied comprehensively, and human studies need to be supplemented and improved.

## Data Availability

The raw data supporting the conclusions of this article will be made available by the authors, without undue reservation.
